# Morphological re-description of *Kurisakia querciphila* Takahashi, 1960 (Hemiptera, Aphididae, Thelaxinae) from South Korea, with notes on its Korean record

**DOI:** 10.3897/BDJ.14.e210973

**Published:** 2026-10-02

**Authors:** Yejin Kang, Hyobin Lee, Wonhoon Lee

**Affiliations:** 1 Department of Plant Medicine, Gyeongsang National University, Jinju, Republic of Korea Department of Plant Medicine, Gyeongsang National University Jinju Republic of Korea; 2 Institute of Agriculture & Life Science, Gyeongsang National University, Jinju, Republic of Korea Institute of Agriculture & Life Science, Gyeongsang National University Jinju Republic of Korea

**Keywords:** apterous vivipara, *COI*, host association, *Quercus variabilis*, species checklist

## Abstract

**Background:**

The genus *Kurisakia* Takahashi, 1924 belongs to the subfamily Thelaxinae (Hemiptera, Aphididae) and currently comprises seven valid species worldwide. *Kurisakia querciphila* Takahashi, 1960 was originally described from Japan as a subspecies of *K. onigurumii* and was subsequently recognised as a distinct species. In Korea, Paik recorded *K. onigurumii*, a record that was later referred to *K. querciphila*. Nevertheless, the current National Species List of Korea lists only *K. onigurumii* within the genus *Kurisakia*, whereas *K. querciphila* is not included.

**New information:**

We provide a detailed morphological re-description and illustrations of the apterous viviparous female of *Kurisakia querciphila*, based on recently collected specimens from South Korea. The specimens were collected from *Quercus variabilis* in Jinju-si, Gyeongsangnam-do, in 2024. Host-plant and distributional information is also provided and the *COI* sequence obtained from a single individual was deposited in GenBank. The present study confirms the occurrence of *K. querciphila* in South Korea, based on morphologically identified specimens and provides taxonomic evidence supporting its inclusion in the National Species List of Korea.

## Introduction

The subfamily Thelaxinae (Hemiptera, Aphididae) is a small group comprising four genera and 21 valid species worldwide (Favret 2026). The genus *Kurisakia* Takahashi, 1924 currently comprises seven valid species (Favret 2026). Species of *Kurisakia* are associated primarily with woody plants of Fagaceae and Juglandaceae, although information on their sexual morphs and life cycles remains limited for most species (Favret and Aphid Taxon Community 2026).

*Kurisakia querciphila* Takahashi, 1960 was originally described from Japan as *Kurisakia onigurumii querciphila*, based on specimens collected from *Quercus acutissima* (Takahashi 1960) and was subsequently treated as a distinct species (Zhang and Zhong 1979). In Korea, Paik recorded *K. onigurumii* from *Q. acutissima* and *Castanea crenata* (Paik 1965), but this record was later referred to *K. querciphila* by Favret and Aphid Taxon Community (2026). Nevertheless, the current National Species List of Korea lists only *K. onigurumii* within *Kurisakia*, whereas *K. querciphila* is not included (National Institute of Biological Resources 2019). Thus, the taxonomic treatment of *Kurisakia* in the Korean fauna warrants clarification and updating.

Detailed morphological information and illustrations of *K. querciphila*, based on Korean specimens remain limited. In the present study, we re-describe and illustrate the apterous viviparous female, based on recently collected specimens and document its host plant and geographic distribution. The *COI* sequence obtained from a single individual was deposited in GenBank. Our results confirm the occurrence of *K. querciphila* in South Korea, based on morphologically identified specimens and provide a taxonomic basis for updating the National Species List of Korea.

## Materials and methods

**Sampling and morphological identification**

Colonies of *K. querciphila* Takahashi, 1960 were collected from the undersides of the young leaves of *Quercus variabilis* in Gajwa-dong, Jinju-si, Gyeongsangnam-do, South Korea (35°09'38.47"N 128°05'46.42"E) in 2024. The samples were preserved in 95% alcohol and mounted in Canada balsam on glass slides, following published procedures (Blackman and Eastop 2000). Slide-mounted specimens were examined using a Leica DM3000 LED light microscope and identified as *Kurisakia querciphila* by comparison with the original description (Takahashi 1960). All specimens were deposited in the Institute of Agriculture & Life Science, Gyeongsang National University, South Korea. The following abbreviations are used in morphological features: BL, body length from the head to the end of cauda; Ant., antennae; Ant.I–Ⅴ and Ant.Ⅴb, antennal segments I–Ⅴ and basal part of antennal segment Ⅴ, respectively; PT, processus terminalis; BDAnt.III, basal diameter of antennal segment III; Setae on Ant.Ⅲ, the longest seta on antennal segment III; 2HT, second segment of hind tarsus; HFM, hind femur; HTB, hind tibia; SIPH, siphunculus; Cauda BW, basal width of cauda; and URS, ultimate rostral segment. Siphuncular measurements are expressed as diameters.

**DNA extraction, amplification, sequence alignment and data analyses**

Total genomic DNA was extracted from a single apterous viviparous female using the AccuPrep® Genomic DNA Extraction Kit (Bioneer, Daejeon, South Korea), following the manufacturer’s protocol. The DNA fragments were amplified using AccuPower® PCR PreMix (Bioneer, Daejeon, South Korea). The partial sequence of the mitochondrial *COI* gene was amplified using the primer pair LCO1490 (5’–GGT CAA CAA ATC ATA AAG ATA TTG G–3’) and HCO2198 (5’–TAA ACT TCA GGG TGA CCA AAA AAT CA–3’) (Folmer et al. 1994). We used the following thermal cycle parameters for a 20 μl amplification reaction: an initial denaturation for 5 min at 94°C, followed by 34 cycles of 1 min at 94°C, 1 min at 48°C and 1 min at 72°C and a final extension at 72°C for 5 min. The PCR product was tested by electrophoresis on 1.5% agarose gel and, if a single band was observed, we commissioned Bionics (Seoul, Korea) for purification and sequencing analysis. The sequence was assembled using SeqMan (version 7.0.1, DNASTAR Inc.) and alignment was performed using MEGA version 12 (Kumar et al. 2024). The *COI* sequence generated in this study has been deposited in GenBank under accession number PZ336710.

## Taxon treatments

### Kurisakia querciphila

Takahashi, 1960

50E261A1-9639-5ECA-BCDD-AA473EF93237

Kurisakia onigurumii querciphila Takahashi 1960: 2
Kurisakia querciphila Zhang & Zhong 1979: 50

#### Materials

- **Type status:** Other material. **Occurrence:** catalogNumber: coll#.20240607-001; recordedBy: Yejin Kang; individualCount: 11; sex: female; lifeStage: apterous female; occurrenceStatus: present; associatedOccurrences: host: Quercus variabilis; occurrenceID: FB596FA1-CE4E-5671-BE4A-FF18EE7B9581; **Taxon:** scientificName: Kurisakia querciphila; kingdom: Animalia; phylum: Arthropoda; class: Insecta; order: Hemiptera; family: Aphididae; genus: Kurisakia; taxonomicStatus: accepted; **Location:** country: South Korea; countryCode: KR; stateProvince: Gyeongsangnam-do; municipality: Jinju-si; locality: Gajwa-dong; verbatimLatitude: 35.160686; verbatimLongitude: 128.096228; geodeticDatum: WGS84; georeferenceProtocol: label; **Identification:** identifiedBy: Yejin Kang; dateIdentified: 2024; **Event:** samplingProtocol: hand collected; year: 2024; month: 6; day: 7; **Record Level:** type: specimen; language: en; rightsHolder: Gyeongsang National University; collectionID: GNU; collectionCode: Insects; basisOfRecord: PreservedSpecimen

#### Description

**Figure 1.**
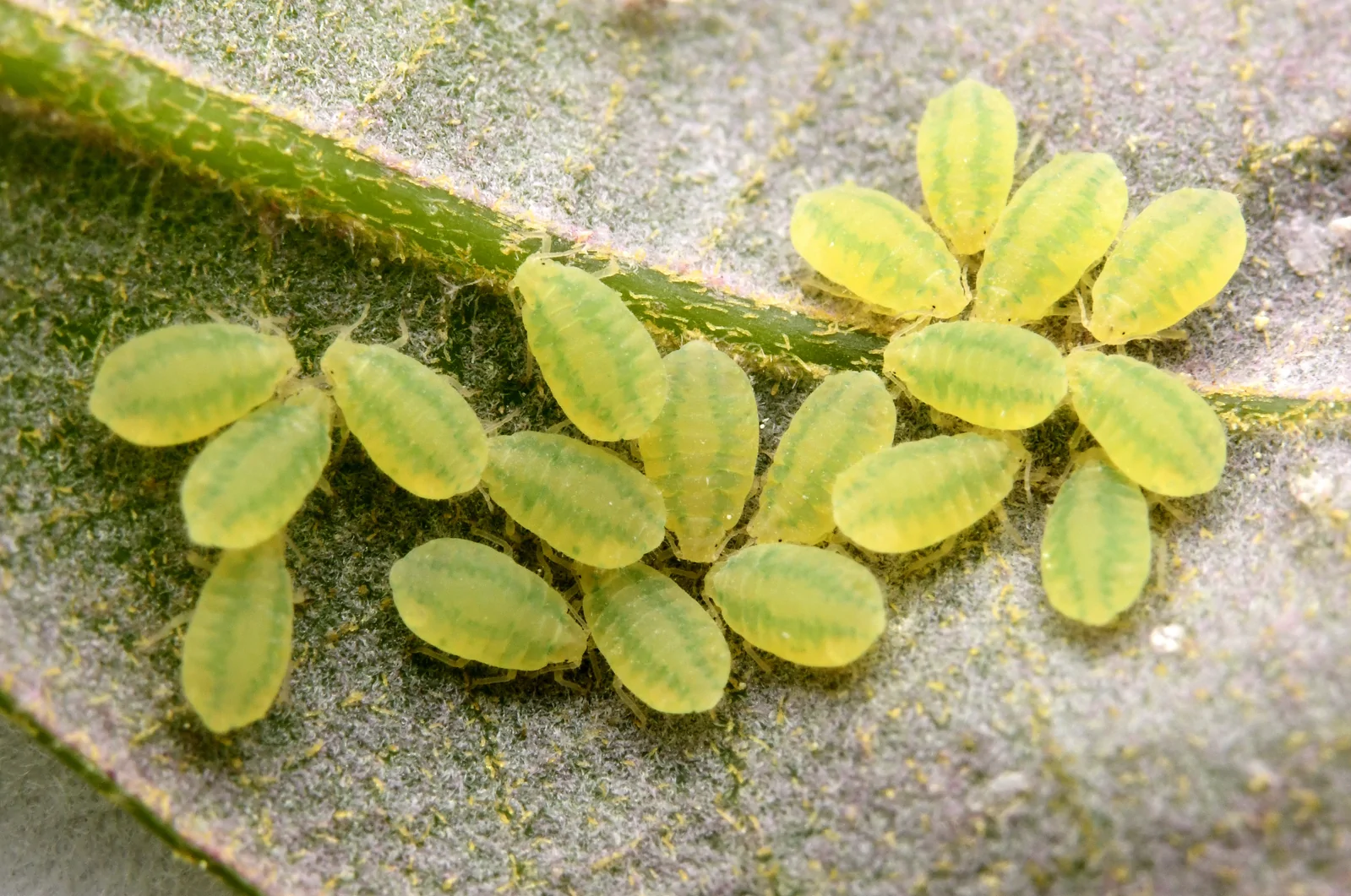
Apterous viviparous females of *Kurisakia querciphila* on *Quercus variabilis*.

**Figure 2.**
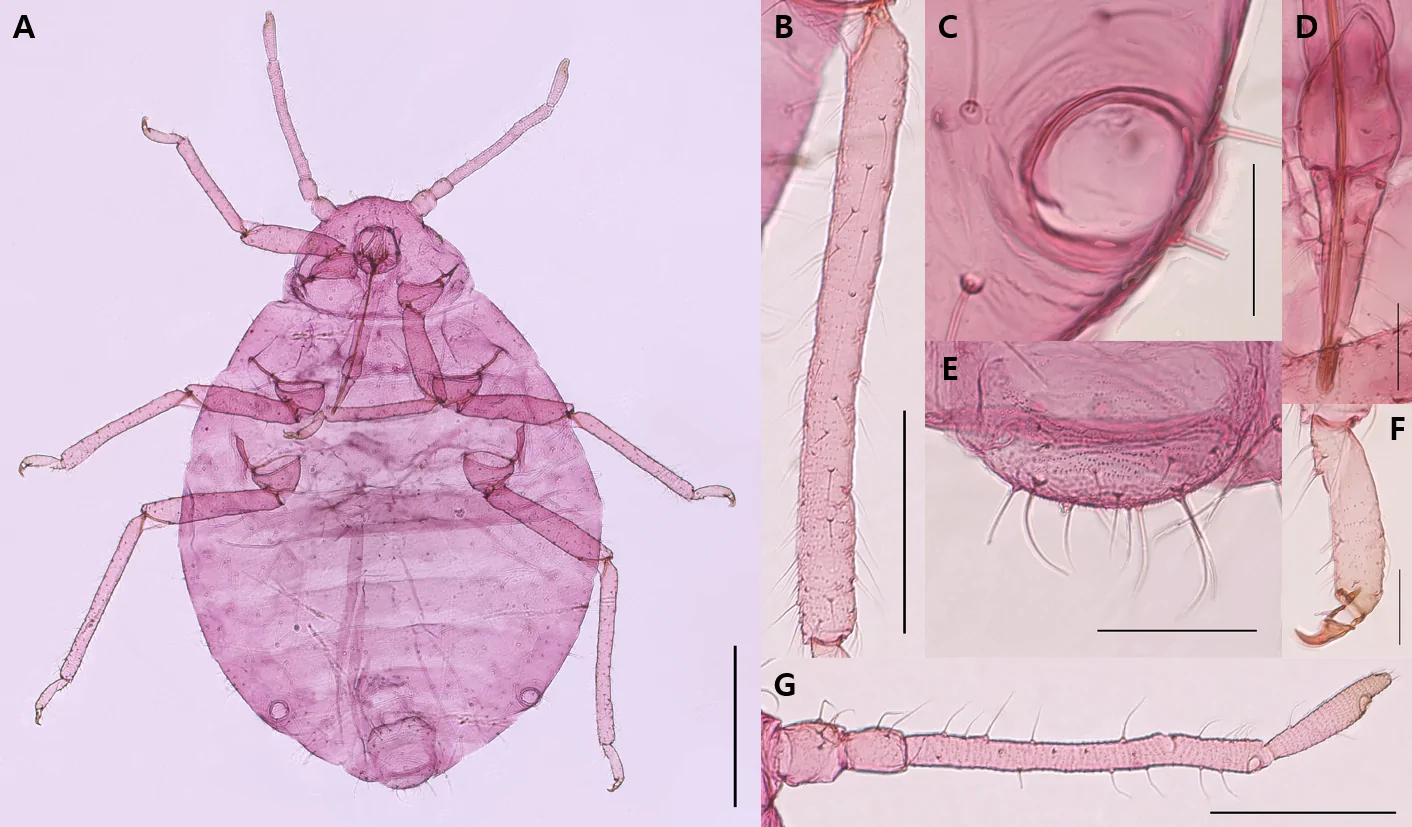
Apterous viviparous female of *Kurisakia querciphila*. **A** Whole body; **B** HTB; **C** SIPH; **D** URS; **E** Cauda; **F** 2HT; **G** Whole Antenna. Scale bar: 500 µm (A); 200 µm (B, G); 100 µm (E); 50 µm (C, D, F).

Apterous viviparous female (Table 1). Body pale yellowish-green with a pair of green vertical streaks on dorsum, antennae; brown PT and tarsus in life (Fig. 1). On mounted specimens, the body is pale brown and oval, 1.82-1.99 mm long (Fig. 2A). Head and prothorax fused; the longest seta on dorsal of the head 1.90-2.67 times as long as middle width of Ant.Ⅲ. Eyes of 3 facets. Antennae 5-segmented, pale brown, except the distal end of Ant.Ⅳ, the half of Ant.Ⅴ is brown; 0.64-0.73 mm long (Fig. 2G). Ant.Ⅰ-Ⅱ weakly spinulose, Ant.Ⅲ with 12-19 setae, mostly spinulose; Ant.Ⅳ and Ant.Ⅴ intensely spinulose with 4-7 setae and 2-4 setae on Ant.Ⅴb; PT not narrowed and 0.04 mm long, with 4-5 short apical setae; 0.31-0.41 times as long as Ant.Ⅴb. The longest seta on antennal segment III is 0.03-0.07 mm long. URS tapering and narrow, 0.12-0.14 mm long, with 5-8 accessory setae; the ultimate rostral segment passing the 2^nd^ coxae (Fig. 2D). Hind femur fused with trochanter, smooth dorsally, spinulose and bearing setae ventrally; hind tibia is spinulose (Fig. 2B); First tarsal segment with 7–8 setae; 2HT brown and spinulose with 7-11 setae (Fig. 2F). Abdomen bearing transverse lines; smooth ventrally, pointed setae dorsally. Siphunculus 0.05-0.07 mm in diameter (Fig. 2C); Cauda with 8-16 (mean 12) setae (Fig. 2E); 0.25-0.36 times as long as width of Cauda.

#### Distribution

South Korea, Japan (Favret and Aphid Taxon Community 2026, Takahashi 1960).

#### Host plants

*Quercus variabilis* (in this study), *Q. acutissima*, *Q. glauca*, *Q. serrata* (Takahashi 1960, Favret and Aphid Taxon Community 2026).

#### Taxonomic remarks

Apterous viviparous females of *K. querciphila* can be distinguished from those of *K. onigurumii* by the number of setae on the first tarsal segment: 7–8 in *K. querciphila* versus 5–6 in *K. onigurumii* (Takahashi 1960). The presence of 7–8 setae on the first tarsal segment was confirmed in the Korean specimens examined in this study.

Published accounts describe apterous females of *K. querciphila* as yellow with paired longitudinal green stripes on the dorsum and those of *K. onigurumii* as green with pale legs and antennae. Their reported host associations also differ: *K. querciphila* is associated with *Quercus*, whereas *K. onigurumii* is principally recorded from *Juglans* and *Pterocarya* (Favret and Aphid Taxon Community 2026). The live colouration of the Korean specimens (Fig. 1) and their occurrence on *Q. variabilis* are consistent with the published account of *K. querciphila*.

Paik’s (1965) Korean record of *K. onigurumii* was subsequently referred to *K. querciphila* (Favret and Aphid Taxon Community 2026). The present study, therefore, provides a detailed morphological re-description, based on newly-collected Korean specimens rather than the first national record of the species.

The *COI* sequence was generated from a single individual and is provided as a reference associated with the morphologically identified material. Molecular sampling was limited to one individual because the aim was to provide a reference sequence, rather than to assess genetic variation within the colony or population. Intraspecific *COI* variation was not assessed in this study.

## Supplementary Material

XML Treatment for Kurisakia querciphila

## Figures and Tables

**Table 1.** The biometric data of *Kurisakia querciphila*.

Body parts		*Kurisakia querciphila* (n = 11)	
		Mean	Range
Measurements (mm)	Body	1.87	1.82–1.99
	Whole antennae	0.68	0.64–0.73
	Ant.Ⅰ	0.07	0.06–0.08
	Ant.Ⅱ	0.07	0.06–0.08
	Ant.Ⅲ	0.29	0.27–0.32
	Ant.Ⅳ	0.11	0.10–0.12
	Ant.Ⅴb	0.11	0.11–0.12
	PT	0.04	0.04–0.04
	URS	0.13	0.12–0.14
	HFM	0.42	0.41–0.43
	HTB	0.54	0.51–0.57
	2HT	0.12	0.11–0.12
	SIPH diameter	0.06	0.05–0.07
	Cauda	0.05	0.05–0.07
	Cauda BW	0.18	0.17–0.20
	BDAnt.Ⅲ	0.04	0.03–0.06
	Setae on Ant.Ⅲ	0.06	0.03–0.07
No. of setae on	Ant.Ⅰ	3	3–4
	Ant.Ⅱ	2	2–3
	Ant.Ⅲ	14	12–19
	URS	7	5–8
	Cauda	12	8–16
Ratio (times)	Whole Ant/BL	0.36	0.33–0.38
	PT/Ant.Ⅴb	0.34	0.31–0.41
	PT/Ant.Ⅲ	0.14	0.12–0.16
	URS/2HT	1.15	1.01–1.29
	URS/Ant.Ⅴb	1.19	1.01–1.28
	HFM/Ant.Ⅲ	1.44	1.32–1.55
	SIPH diameter/BL	0.03	0.03–0.04
	SIPH diameter/Ant.Ⅲ	0.20	0.17–0.22
	Cauda/Cauda BW	0.30	0.25–0.36
	Setae on Ant.Ⅲ/BDAnt.Ⅲ	1.71	0.62–2.11
